# Structure–Activity Relationship of Novel Second-Generation Synthetic Cathinones: Mechanism of Action, Locomotion, Reward, and Immediate-Early Genes

**DOI:** 10.3389/fphar.2021.749429

**Published:** 2021-10-26

**Authors:** Nuria Nadal-Gratacós, Ana Sofia Alberto-Silva, Míriam Rodríguez-Soler, Edurne Urquizu, Maria Espinosa-Velasco, Kathrin Jäntsch, Marion Holy, Xavier Batllori, Xavier Berzosa, David Pubill, Jordi Camarasa, Harald H. Sitte, Elena Escubedo, Raúl López-Arnau

**Affiliations:** ^1^ Department of Pharmacology, Toxicology and Therapeutic Chemistry, Faculty of Pharmacy, Pharmacology Section and Institute of Biomedicine (IBUB), University of Barcelona, Barcelona, Spain; ^2^ Pharmaceutical Chemistry Group (GQF), IQS School of Engineering, Universitat Ramon Llull, Barcelona, Spain; ^3^ Center for Physiology and Pharmacology, Institute of Pharmacology, Medical University Vienna, Vienna, Austria; ^4^ Center for Addiction Research and Science, Medical University Vienna, Vienna, Austria

**Keywords:** synthetic cathinones, new psychoactive substance, psychostimulant, reward, immediate-early gene (IEG), structure–activity relationship, releasers

## Abstract

Several new synthetic cathinones, which mimic the effect of classical psychostimulants such as cocaine or MDMA, have appeared in the global illicit drug market in the last decades. In fact, the illicit drug market is continually evolving by constantly adding small modifications to the common chemical structure of synthetic cathinones. Thus, the aim of this study was to investigate the *in vitro* and *in vivo* structure–activity relationship (SAR) of six novel synthetic cathinones currently popular as recreational drugs, pentedrone, pentylone, N-ethyl-pentedrone (NEPD), N-ethyl-pentylone (NEP), 4-methyl-pentedrone (4-MPD), and 4-methyl-ethylaminopentedrone (4-MeAP), which structurally differ in the absence or presence of different aromatic substituents and in their amino terminal group. Human embryonic kidney (HEK293) cells expressing the human isoforms of SERT and DAT were used for the uptake inhibition and release assays. Moreover, Swiss CD-1 mice were used to investigate the psychostimulant effect, rewarding properties (3, 10, and 30 mg/kg, i.p.), and the induction of immediate-early genes (IEGs), such as *Arc* and *c-fos* in the dorsal striatum (DS) and ventral striatum (VS) as well as *bdnf* in the medial prefrontal cortex (mPFC), of the test compounds. Our results demonstrated that all tested synthetic cathinones are potent dopamine (DA) uptake inhibitors, especially the N-ethyl analogs, while the ring-substituted cathinones tested showed higher potency as SERT inhibitors than their no ring-substituted analogs. Moreover, unlike NEP, the remaining test compounds showed clear “hybrid” properties, acting as DAT blockers but SERT substrates. Regarding the locomotion, NEP and NEPD were more efficacious (10 mg/kg) than their N-methyl analogs, which correlates with their higher potency inhibiting the DAT and an overexpression of *Arc* levels in the DS and VS. Furthermore, all compounds tested induced an increase in *c-fos* expression in the DS, except for 4-MPD, the least effective compound in inducing hyperlocomotion. Moreover, NEP induced an up-regulation of *bdnf* in the mPFC that correlates with its 5-HTergic properties. Finally, the present study demonstrated for the first time that NEP, 4-MPD, and 4-MeAP induce reward in mice. Altogether, this study provides valuable information about the mechanism of action and psychostimulant and rewarding properties as well as changes in the expression of IEGs related to addiction induced by novel second-generation synthetic cathinones.

## Introduction

During the last decades, the illicit drug market has undergone a diversification of the drugs available, as there has been a dramatic and rapid emergence and propagation of new psychoactive substances (NPSs). Within the categories of NPSs present in the market, one of the main substance classes is formed by the synthetic cathinones, which simulate the effects of traditional psychostimulant drugs such as cocaine, 3,4-methylenedioxymethamphetamine (MDMA), or methamphetamine (Sitte and Freissmuth, 2015). The popularity of synthetic cathinones as recreational drugs has increased since the mid-2000s. However, the illicit drug market is continuously evolving, and a wider variety of new alternatives have emerged by adding constant and small modifications to the common chemical structure of cathinone (Baumann et al., 2013).

**FIGURE 1:**
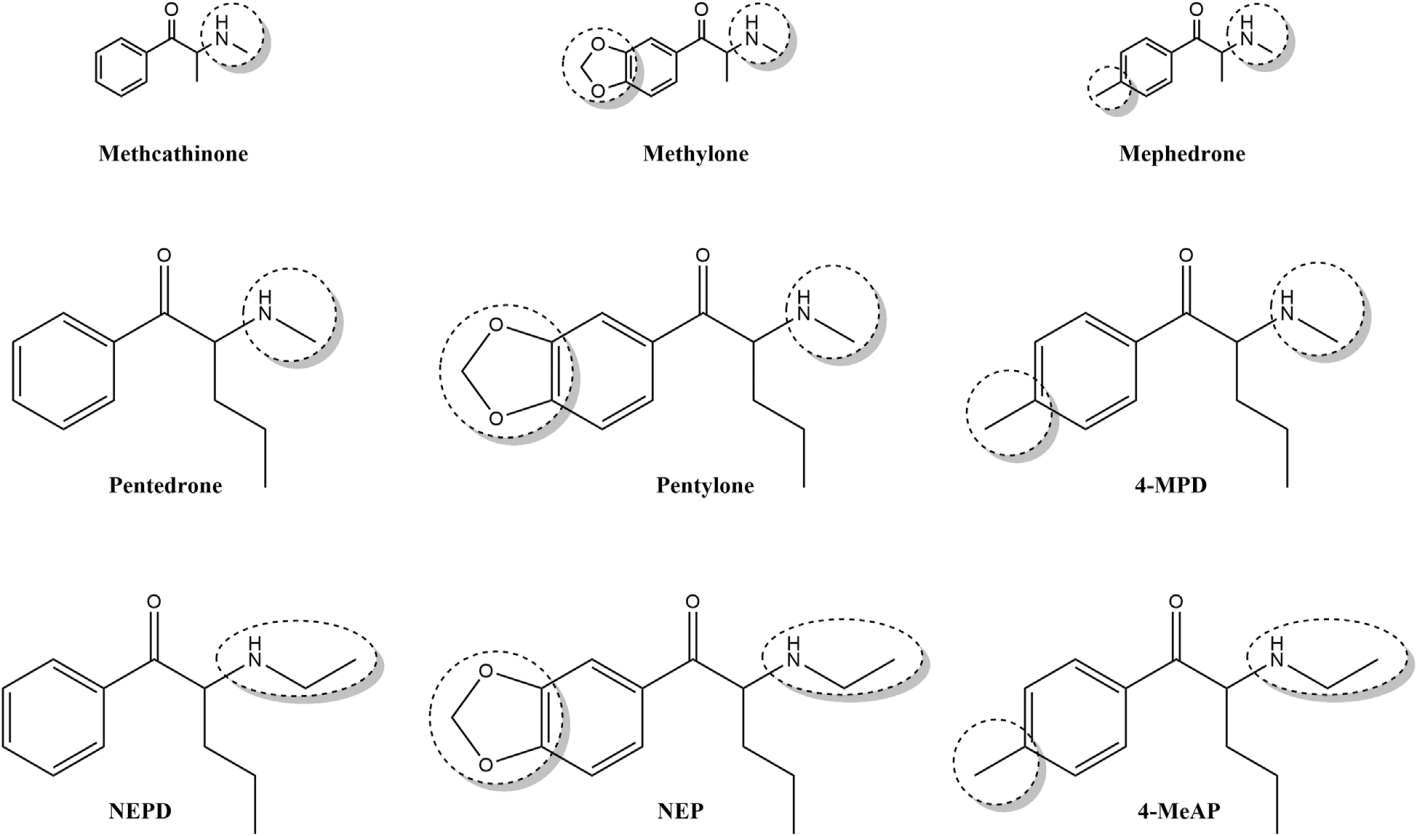
Chemical structure of three first-generation synthetic cathinones (methcathinone, methylone, and mephedrone) and six novel ring-substituted α-aminovalerophenone derivatives (pentedrone, pentylone, 4-MPD, NEPD, NEP, and 4-MeAP).

Pentylone, 4-methyl-pentedrone (4-MPD), and N-ethyl-pentylone (NEP) (Figure 1) were among the 10 most frequently reported synthetic cathinones (DEA, 2016; DEA, 2018). Specifically, N-ethyl-pentylone (NEP) has been identified as one of the most recent novel stimulants to emerge into the arena of evolving NPSs (Krotulski et al., 2018) and accounted for approximately 62% of cathinone identifications, being the most reported synthetic cathinone in 2018 (DEA, 2018). Moreover, some intoxications and even fatalities associated with pentylone, 4-MPD, NEP, N-ethyl-pentedrone (NEPD), and 4-methyl-ethylaminopentedrone (4-MeAP) use have been recently reported (Fujita et al., 2015; Varma et al., 2017; Majchrzak et al., 2018; Zaami et al., 2018; Benedicte et al., 2020; Giachetti et al., 2020; Lau et al., 2020; Weng et al., 2020; Cartiser et al., 2021). Regarding the mechanism of action, synthetic cathinones are able to competitively inhibit dopamine (DA) and serotonin (5-HT) uptake (López-Arnau et al., 2012; Simmler et al., 2014; Duart-Castells et al., 2021); for a review, see Riley et al. (2020). However, it has been demonstrated that pentylone has hybrid activity, acting as a DA transporter (DAT) blocker but a 5-HT transporter (SERT) substrate (Saha et al., 2019). In fact, it is widely known that their chemical structure can impact their potency, selectivity, mechanism of action, and *in vivo* effects (Coppola and Mondola, 2012; Kolanos et al., 2013; Gonçalves et al., 2019; Walther et al., 2019); for a review, see Glennon and Dukat (2017).

Thus, the present study is focused on six novel synthetic cathinones: pentedrone, pentylone, NEPD, NEP, 4-MPD, and 4-MEAP, which structurally differ in the absence or presence of different aromatic substituents (4-methyl- and 3,4-methylenedioxy-substitution) as well as in the amino terminal group (methyl- vs ethyl-substitution). Furthermore, these cathinones were also chosen for their recent appearance in the illicit drug market and their current popularity as recreational drugs.

Injection of several drugs of abuse has been demonstrated to cause changes in the expression of immediate-early genes (IEGs) which codify for inducible transcription factors which play a role in the transition from a recreational to a compulsive drug use (Lanahan and Worley, 1998). However, little is known about the changes in the expression of immediate-early genes induced by several novel synthetic cathinones. Among these IEGs, our study is focused on 1) *c-fos*, a neuronal activity marker whose expression indicates a response of adaptation, memory formation, and neuroplasticity (Kovács, 2008; Gallo et al., 2018), 2) *Arc* (activity-regulated cytoskeleton-associated protein), involved in neuronal plasticity procedures that follow dopaminergic activity (Kovács, 2008; Gallo et al., 2018), and 3) *bdnf* (brain-derived neurotrophic factor) implicated in the neuroadaptations that manage lasting functional changes in neuronal synapses (Russo et al., 2009; Ghitza et al., 2010; McGinty et al., 2010). There is convincing evidence that BDNF, along with its specific receptor (TrkB), has a key role in the behavioral abnormalities observed in rodents after psychostimulant administration; for a review, see Li and Wolf (2015). The main brain area involved in the effect of psychostimulants is the nucleus accumbens (NAcc), which is a major component of the ventral striatum (VS). Therefore, a relationship can be observed between the effectivity of a psychostimulant and an increase of *c-fos* expression in the VS*.* The dorsal striatum (DS) is also a brain region very rich in dopaminergic terminals. Thus, drugs that enhance significantly the dopaminergic activity must also increase *c-fos* and *Arc* expressions in these rich dopaminergic areas (Sheng et al., 1990; Banerjee et al., 2009). On the contrary, the BDNF present in the NAcc and striatum is chiefly supplied by anterograde axonal transport from cortical pyramidal neurons in the medial prefrontal cortex (mPFC) (Altar et al., 1997; Conner et al., 1997).

Therefore, the aim of the present study was, firstly, to synthetize and characterize these six novel synthetic cathinones as previously outlined. Secondly, we aimed to study the *in vitro* neuropharmacological properties of these compounds (interactions with the DAT and SERT) and, finally, to behaviorally investigate not only their *in vivo* psychostimulant and rewarding effects but also the expression of IEGs related to drug addiction, after an acute administration in mice. Altogether, this study may provide a detailed structure–activity relationship (SAR) between novel synthetic cathinones, their mechanism of action, and a characterization of their psychostimulant and rewarding properties. Moreover, the analysis of *c-fos*, *Arc*, and *bdnf* mRNA expressions in different brain areas after acute administration of these novel synthetic cathinones could provide us with valuable information about the neuroadaptive processes in the brain circuitry, which are initiated by changes in the transcription of IEGs.

## Materials and Methods

### Subjects

For the behavioral and gene expression experiments, male Swiss CD-1 mice (Janvier, Le Genest, France) weighing 30–35 g (6–8 weeks old) were used. With the aim of reducing the number of animals and minimize their suffering, animal procedures were performed in accordance with the ARRIVE guidelines. All animal care and experimental protocols in this study were in accordance with the guidelines of the European Community Council (2010/63/EU), as amended by Regulation (EU) 2019/1010, and approved by the Animal Ethics Committee of the University of Barcelona under the supervision of the Autonomous Government of Catalonia. The animals were housed in temperature-controlled conditions (22 ± 1°C) under a 12 h light/dark cycle and had free access to food and drinking water. During the study, the animals were supervised immediately after injection and during the behavioral procedure as well as 24 h after injection, and different parameters were visually evaluated for humane endpoints, such as self-mutilations, strange vocalizations, abnormal posture, or a greater weight loss than 20%. The sample size was determined using GPower software. The minimal significance (α) was set at 0.05 and statistical power at 0.8.

### Drugs and Materials

Ring-substituted α-aminovalerophenone derivatives were synthetized in racemic form as hydrochloride salts as described in the Supplemental Material. Chemical purity and identification of the synthetic cathinones was assessed by thin-layer chromatography (TLC), proton and carbon nuclear magnetic resonance (^1^H NMR, ^13^C NMR), infrared spectroscopy (IR), and mass spectrometry (MS). Solutions for injection were prepared daily in isotonic saline solution (0.9% NaCl, pH 7.4). Cocaine was provided by the Spanish National Institute of Toxicology. [^3^H]1-Methyl-4-phenylpyridinium ([^3^H]MPP^+^) was supplied by American Radiolabeled Chemicals (St. Louis, United States). [^3^H]5-HT was purchased from Perkin Elmer, Inc. (Boston, MA, United States). All other reagents were of analytical grade and purchased from several commercial sources.

### Uptake Inhibition Assays in HEK293 Cells

#### Cell Culture

Human embryonic kidney (HEK293) cells stably expressing the yellow-fluorescent protein (YFP)-tagged version of the human isoforms of SERT (abbreviated as SERT) and DAT (abbreviated as DAT) were used for the uptake inhibition and release assays. The generation and maintenance of stable, monoclonal cell lines expressing these transporters was conducted as described by (Mayer F. P. et al., 2016; Mayer et al., 2016 FP.). HEK293 cells were maintained in DMEM supplemented with heat-inactivated 10% FBS, 100 µg/100 ml streptomycin, 100 U/ml penicillin, and Geneticin (G418; 50 μg/ml) and cultured in a humidified atmosphere (5% CO_2_, 37°C). The cells were seeded at a density of 0.36 million cells/well onto 96-well plates previously coated (24 h) with poly-D-lysine (PDL).

#### Uptake Inhibition Assays

The medium was removed and immediately replaced with 200 µL/well of Krebs-HEPES buffer (KHB; 10 mM HEPES, 120 mM NaCl, 3 mM KCl, 2 mM CaCl_2_·2H_2_O, 2 mM MgCl_2_·6H_2_O, 20 mM D-glucose; pH 7.3). The cells were pre-incubated with different concentrations of the drug in KHB at a final volume of 50 µL/well for 5 min (DAT, SERT). Immediately after, the pre-incubation solution was removed and the cells were incubated with the tritiated substrates, 0.02 µM [^3^H]MPP^+^ for the DAT (3 min) and 0.1 µM [^3^H]5-HT for the SERT (1 min), along with different concentrations of the drug in KHB. Finally, the [^3^H]substrate was removed and the cells were washed with ice-cold KHB followed by the addition of sodium dodecyl sulfate (SDS) 1%. The lysate was then added to the scintillation fluid, and the radioactivity was quantified with a beta-scintillation counter (Perkin Elmer, Waltham, MA, United States).

Non-specific uptake was determined in parallel samples containing cocaine 100 µM for the HEK293-DAT cell line and paroxetine 3 µM for the HEK293-SERT cell line. The non-specific uptake represented <10% of total uptake. The uptake in the absence of the drug compound was normalized to 100%, being the percentage of the uptake in the presence of different concentrations of the drug expressed as a percentage thereof. 3–4 independent experiments were performed in duplicate.

#### Release Assays

HEK293 cells expressing the transporter of interest were preloaded with the tritiated substrate 0.08 μM [^3^H]5-HT (SERT) or 0.1 μM [^3^H]MPP^+^ (DAT) in KHB (50 μL/well) for 20 min (5% CO_2_, 37°C). Subsequently, the cells were washed three times using KHB (200 μL/well) at room temperature. Afterward, a pre-incubation step of 10 min in KHB or in KHB + monensin (Mon) 10 μM was done. Finally, the synthetic cathinone of interest (in KHB or in KHB + Mon, 100 μL/well) was added at the corresponding IC_50_ or at 10 μM (only in experiments involving the DAT). To determine the specificity of drug-induced reverse transport, selective transporter inhibitors 0.05 μM paroxetine (SERT) and 0.5 μM of GBR12909 (DAT) and effective releasers 10 μM *p*-chloroamphetamine (PCA; SERT) and 10 μM *d*-amphetamine (DAT) were used (Supplementary Figure S1). The resulting supernatant was collected and transferred to a new well every 2 min (four times for KHB/Mon and five times for each tested compound). Three independent experiments were performed in duplicate. Liquid scintillation cocktail was added to the wells with remaining cells (200 μL) and with the transferred supernatant (100 μL) and to the wells used for total uptake and activity measurements (200 μL). Total radioactivity present in the supernatant and in the remaining cells was set as 100%, and the amount of [^3^H] substrate present in the supernatant was expressed as percentage of the total.

### Horizontal Locomotor Activity

An habituation phase was previously performed, in which all mice received, for two consecutive days, an intraperitoneal (i.p.) saline injection and were immediately placed into a black Plexiglas arena (25 × 25 × 40 cm) under low-light conditions and white noise for 30 min. On the test day, mice received i.p. injections of saline or different doses (3, 10, or 30 mg/kg) of the drug and then were immediately placed into the same arena under the same conditions of light and noise. The HLA was video-monitored for 1 h using a specific tracking software program (Smart 3.0, Panlab, Spain), and the total traveled distance (in cm) was measured. The doses were chosen according to the psychostimulant effect induced by pentedrone and NEPD in previous studies (Duart-Castells et al., 2021). Human doses of pentedrone are reported to be variable. In fact, the WHO critical review report of 2016 regarding pentedrone human doses reports 80–150 mg by oral route, 40–100 mg by nasal route, and 30–60 mg by intravenous route (dosage range of 1.2–2.3 mg/kg by oral route, 0.6–1.5 mg/kg by nasal route, and 0.5–0.9 mg/kg by intravenous route for a 65 kg person) (World Health Organization (WHO), 2016). According to the method of conversion of animal dose to human-equivalent dose using the normalization by body surface area (mg/m^2^), 3, 10, and 30 mg/kg in mice correspond approximately to human doses of 15, 51, and 154 mg (0.2, 0.8, and 2.4 mg/kg approximately for a 65 kg person) (Reagan-Shaw et al., 2008; Sharma and McNeill, 2009). As can be observed, the range of doses used in this study is in accordance with the dosage reported by consumers. However, it must be pointed out that the dose of 30 mg/kg is still a high dose, but we cannot rule out the possibility that synthetic cathinones are taken as intermittent binges, increasing significantly their plasma concentration. Moreover, it is important to study the effects of cathinones at high doses in order to have a wider understanding of the effects.

### Conditioned Place Preference

The rewarding effects were determined using a place conditioning paradigm as described by Duart-Castells et al. (2019). Briefly, the apparatus consists of two different (visual and tactile) compartments communicated by a corridor. In the preconditioning phase (Day 0), mice were placed in the middle of the corridor and had free access to both compartments for 15 min. The time spent in each compartment was recorded (Smart 3.0, Panlab, Spain). During the conditioning phase (Days 1–4; two sessions/day), mice received an i.p. injection (3, 10, or 30 mg/kg) of the corresponding drug or saline (alternate sessions separated by a 5 h period) and were immediately placed into one of the compartments for 20 min. Control groups received a saline injection in every session. Sessions were counterbalanced as much as possible between compartments.

Finally, the post-conditioning test (Day 5) was performed as the preconditioning phase. A preference score was calculated as the difference between the time spent in the drug-paired compartment in the post-conditioning test and the time spent in the preconditioning phase.

### Tissue Sample Preparation, RNA Extraction, and Gene Expression Determination

Mice were injected with an acute dose of 10 mg/kg i.p. of the corresponding synthetic cathinone or saline solution. The dose was chosen according to the behavioral results obtained since it caused significant response in both CPP and HLA tests in all compounds tested. 30 min or 2 h post-administration, mice were sacrificed by cervical dislocation and the VS, DS, and mPFC were dissected out (Paxinos and Franklin, 2004) using a mouse brain acrylic matrix (Agnthos, Sweden) and stored at -80°C until use. Total RNA was isolated following a standard TRIsure reagent–chloroform extraction protocol (Duart-Castells et al., 2019).

Reverse transcription polymerase chain reaction (RT-PCR) was carried out to obtain complementary DNA (cDNA) by using the High Capacity cDNA Reverse Transcription Kit (Applied Biosystems) and the Veriti thermal cycler (Applied Biosystems, Foster, CA, United States). Synthesis of cDNA was obtained by mixing 1,000 ng of total RNA with the corresponding volumes of each kit reagent to a final volume of 20 μL. Quantitative real-time polymerase chain reaction (qPCR) was performed by using the StepOnePlus^TM^ Real-Time PCR System and TaqMan PCR Master Mix (Applied Biosystems, Foster, CA, United States). 25 ng of cDNA was loaded in a final volume of 20 μL containing sequence-specific primers and TaqMan probes (Mm01204954_g1 for *Arc*, Mm04230607_s1 for *bdnf*, Mm00487425_m1 for *c-fos*, and Mm00607939_s1 for *β-actin*). Each sample was tested in duplicate. qPCR conditions were 2 min at 50°C, 10 min at 95°C, 40 cycles/15 s at 95°C, and 60 s at 60°C. Changes in gene expression were defined by using the comparative Ct method for each experimental sample. Gene expression was normalized by using the mean of Ct values of the housekeeping gene *β-actin*.

### Data Analysis

Data were expressed as mean ± SEM. Competition curves were plotted and fitted by non-linear regression, and data were best fitted to a sigmoidal dose-response curve to obtain an IC_50_ value. 1/DAT IC_50_: 1/SERT IC_50_ was used to calculate transporter ratios. Data from batch release assays were statistically analyzed with a mixed-effects model, employing Šidák’s correction for multiple comparisons. One-way/two-way ANOVA or two-way ANOVA of repeated measures, followed by Tukey’s or Dunnett’s post hoc test, was also used when appropriate. The α error probability was set at 0.05 (*p* < 0.05). The size group for each experiment and statistical results are shown in the corresponding legend of figures. Statistical calculations were performed using GraphPad Prism (GraphPad Software, San Diego, CA, United States).

## Results

### Effect on *In Vitro* Monoamine Uptake Inhibition and Release Assays

**FIGURE 2:**
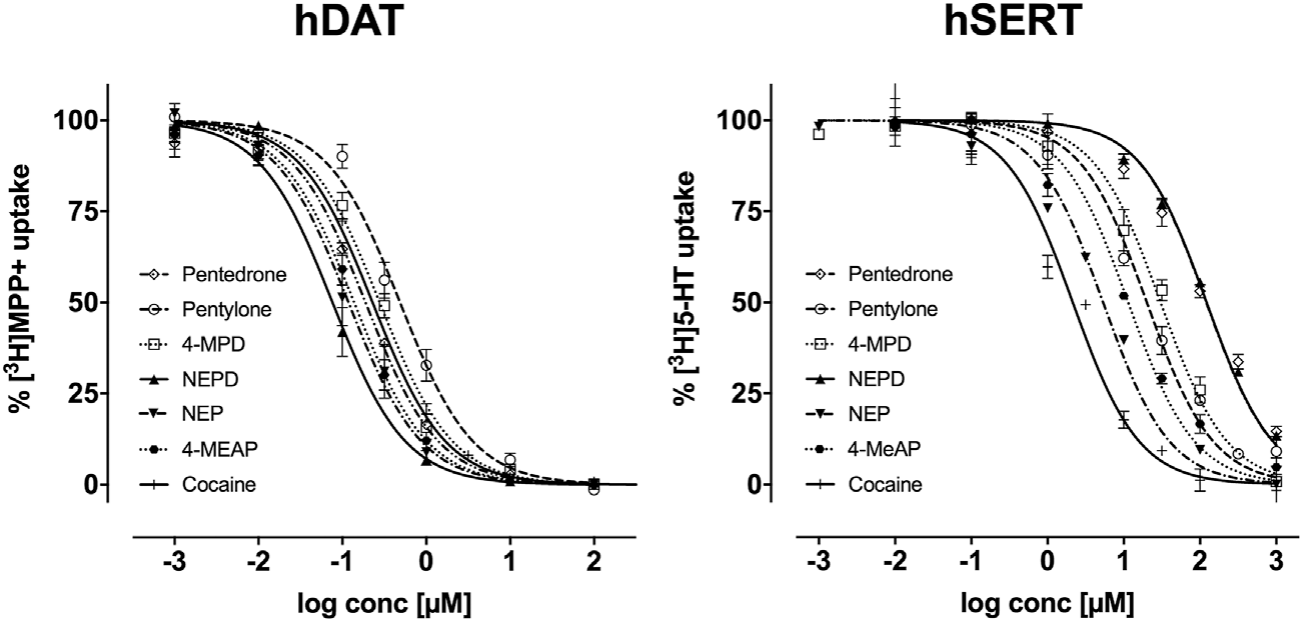
Effects of pentedrone, pentylone, 4-MPD, NEPD, NEP, and 4-MeAP on [^3^H]MPP^+^ uptake inhibition at the DAT and [^3^H]5-HT uptake inhibition at the SERT in transfected HEK293 cells. Data are expressed as a percentage of control uptake (mean ± SEM) of 3-4 independent experiments performed in triplicate.

The IC_50_ values obtained in monoamine uptake inhibition assays and the resulting DAT/SERT ratios are compiled in Table 1. Concentration-response curves are depicted in Figure 2. All tested synthetic cathinones presented low activity at the SERT together with a high potency inhibiting [^3^H]MPP^+^ uptake resulting in high DAT/SERT ratio values. For both the DAT and SERT, the capacity to inhibit [^3^H]MPP^+^ or [^3^H]5-HT uptake increased with the presence of ethyl amine (NEPD > pentedrone; NEP > pentylone; 4-MeAP > 4-MPD), being this effect stronger on the DAT. Regarding [^3^H]5-HT uptake on the SERT, pentedrone and NEPD showed a much lower inhibition potency than the rest of the tested drugs. On the contrary, NEP was the substance with a highest inhibitory potential toward this transporter (IC_50_ = 6.37 ± 0.16 µM).

**TABLE 1:** Potency of substituted cathinones and standard compounds at monoamine transporters. Monoamine uptake inhibition: values are *IC*_*50*_ given in µM (mean ± SEM).

	Monoamine uptake inhibition		
	Transfected HEK293 cells		
	Uptake-1		
Compound	[^3^H]MPP^+^ uptake at hDAT	[^3^H]5-HT uptake at hSERT	hDAT/hSERT ratio
Pentedrone	0.21 ± 0.02	137.9 ± 13.4	666
Pentylone	0.51 ± 0.07	23.2 ± 2.68	45
4-MPD	0.29 ± 0.05	30.88 ± 6.23	108
N-Ethyl-pentedrone	0.10 ± 0.03	127.1 ± 5.14	>1,000
N-Ethyl-pentylone	0.13 ± 0.01	6.37 ± 0.09	51
4-MeAP	0.14 ± 0.02	13.27 ± 0.89	93
Cocaine^a^	0.23 ± 0.01	1.82 ± 0.10	7.84

hDAT/hSERT ratio = 1/DAT IC_50_: 1/SERT IC_50_.

**FIGURE 3:**
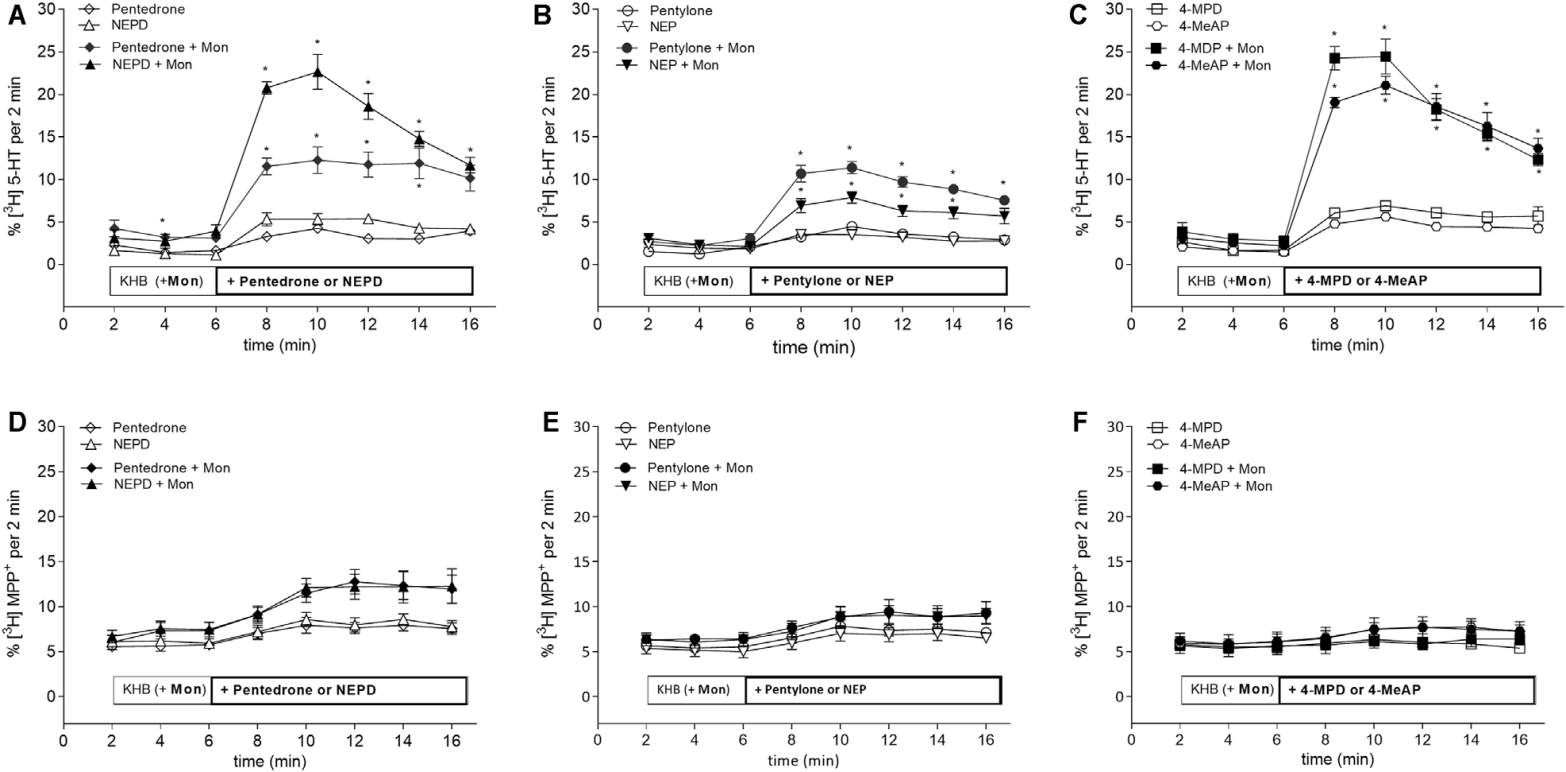
Effects of pentedrone, pentylone, 4-MPD, NEPD, NEP, and 4-MeAP on transport-mediated release of preloaded radiolabeled substrate from HEK293 cells stably expressing the **(A–C)** SERT or **(D–F)** DAT. As indicated, after 6 min of basal release (in the presence of either KHB or KHB with monensin (Mon) 10 µM), the compound of interest was added at a concentration close to its predetermined IC_50_ value for each transporter (SERT: pentedrone and NEPD (130 µM), pentylone (20 µM), NEP (6 µM), 4-MPD (30 µM), and 4-MeAP (10 µM); DAT: pentedrone and pentylone (0.5 µM), NEPD (0.2 µM), NEP and 4-Me-AP (0.1 µM), and 4-MPD (1 µM)). Synthetic cathinones were grouped in pairs according to the presence of a methyl or ethyl group at the amino terminal (pentedrone and NEPD; pentylone and NEP; 4-MPD and 4-MeAP). * denotes *p* < 0.05.

Regarding release assays, NEPD, 4-MPD, and 4-MeAP have been shown to be the most potent synthetic cathinones evoking [^3^H]5-HT release at the SERT close to their determined IC_50_ concentrations. Followed by the latter, pentedrone and pentylone evoked milder release at the SERT. Finally, NEP evoked the smallest [^3^H]5-HT release at SERT-expressing HEK293 cells (max.: ≈5% total cpm) (Figure 3). Data were statistically analyzed using a mixed-effects model, employing Šidák’s correction for multiple comparisons. This statistical analysis explored possible significant differences between KHB + compound and KHB with Mon 10 µM + compound at the indicated time points, thus underlining the releasing capabilities of each compound for the SERT (see statistical results in Supplementary Table S1). When compared with the positive and negative controls, all these results suggest that these substances act as partial releasers at the SERT. Moreover, batch release assays have also shown that all these synthetic cathinones have none to negligible effect in releasing [^3^H]MPP^+^ at DAT-expressing HEK293 cells, both when tested at IC_50_ (Figure 3) or 10 µM concentrations (Supplementary Figure S2).

### Effect on Horizontal Locomotor Activity

**FIGURE 4:**
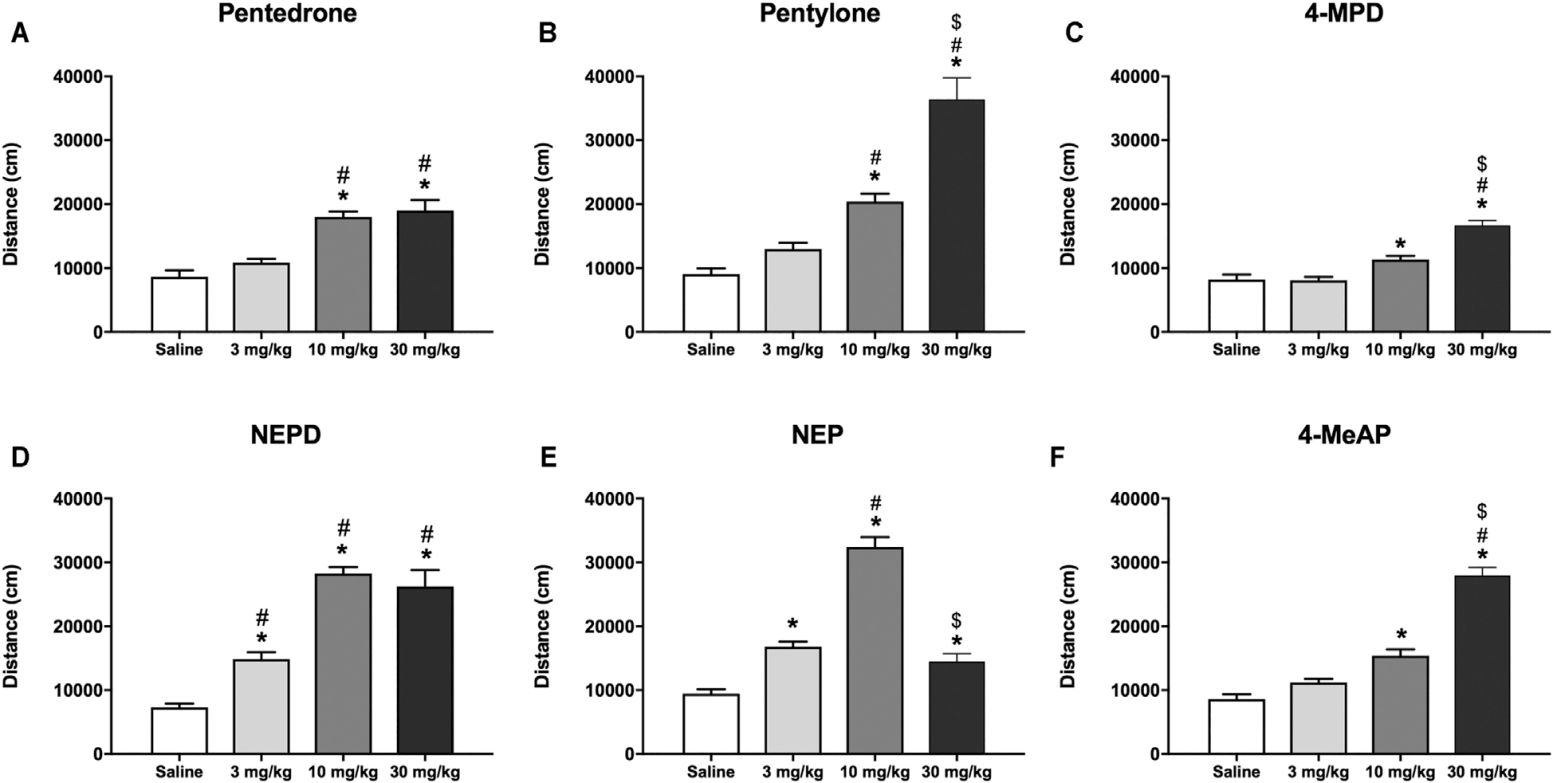
Effects of pentedrone **(A)**, pentylone **(B)**, 4-MPD **(C)**, NEPD **(D)**, NEP **(E)**, and 4-MeAP **(F)** on cumulative HLA in mice. Bars represent the mean ± SEM of the total distance (cm) traveled in 60 min. *Tukey’s* multiple-comparisons test; ^*^ *p* < 0.05 vs the saline group; ^#^ *p* < 0.05 vs the 3 mg/kg dose group; ^$^ *p* < 0.05 vs the 10 mg/kg dose group (*N* = 12–14/group).

As shown in Figures 4A–F, all compounds increased the HLA in a dose-dependent manner in mice. One-way ANOVA of the distance traveled revealed a significant effect of the variable *Dose* for all the compounds tested (pentedrone: F_(3, 44)_ = 22.00, *p* < 0.05; pentylone: F_(3, 44)_ = 39.53, *p* < 0.05; 4-MPD: F_(3, 50)_ = 33.29, *p* < 0.05; NEPD: F _(3, 44)_ = 43.63, *p* < 0.05; NEP: F_(3, 51)_ = 78.22, *p* < 0.05; 4-MeAP: F_(3, 52)_ = 86.89, *p* < 0.05). For all substances, a significant increase in HLA after 10 and 30 mg/kg injections was observed. However, only NEPD and NEP also induced an increase in HLA at the lowest dose tested (3 mg/kg). Moreover, pentedrone and NEPD showed a ceiling effect after 30 mg/kg injection, unlike pentylone, 4-MPD, and 4-MeAP, in which a significant increase was observed after 30 mg/kg injection compared to the medium dose tested (10 mg/kg). By contrast, a significant decrease in HLA was observed after NEP (30 mg/kg) administration compared to the medium dose tested (10 mg/kg), showing an inverted U-shaped profile.

**FIGURE 5:**
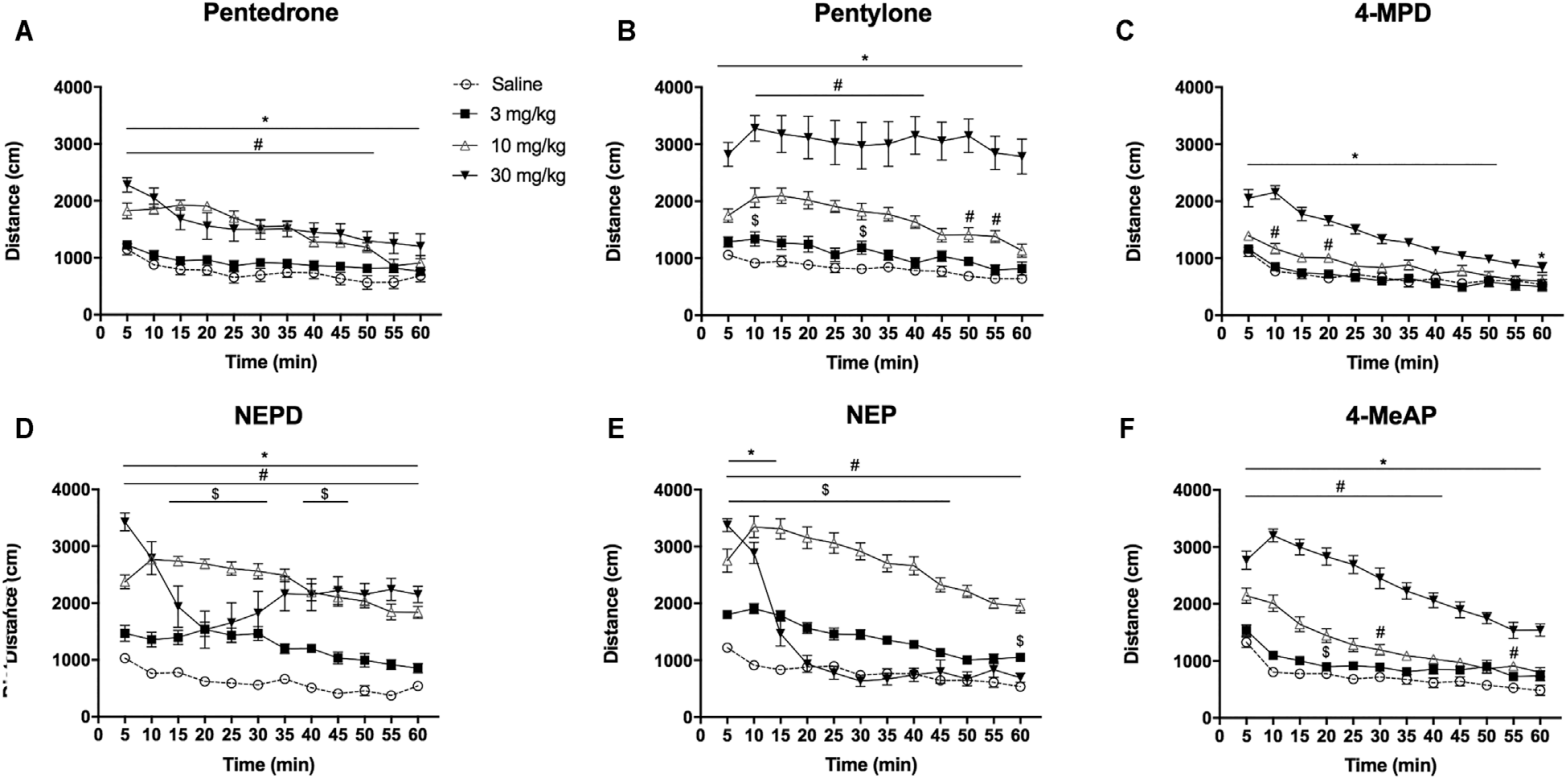
Time course profile of HLA induced by pentedrone **(A)**, pentylone **(B)**, 4-MPD **(C)**, NEPD **(D)**, NEP **(E)**, and 4-MeAP **(F)**. Each time point represents the mean ± SEM of the distance (in cm) traveled in 5 min blocks. Only comparisons vs the corresponding saline group are shown for clarity purposes. *Tukey’s* multiple-comparisons test; ^*^ *p* < 0.05, 30 mg/kg dose group vs saline group; ^#^ *p* < 0.05, 10 mg/kg dose group vs saline group; ^$^ *p* < 0.05, 3 mg/kg dose group vs saline group (*N* = 12–14/group).

HLA time courses are depicted in Figure 5. Two-way ANOVA of repeated measures of the results yielded the following results: pentedrone: *Dose*: F_(3, 44)_ = 20.21, *p* < 0.05; *Time*: F_(11, 484)_ = 29.58, *p* < 0.05; *Interaction*: F_(33, 484)_ = 3.887, *p* < 0.05; pentylone: *Dose*: F_(3, 43)_ = 37.69, *p* < 0.05; *Time*: F_(11, 473)_ = 9.965, *p* < 0.05; *Interaction*: F_(33, 473)_ = 1.590, *p* < 0.05; 4-MPD: *Dose*: F_(3, 52)_ = 30.57, *p* < 0.05; *Time*: F_(11, 572)_ = 78.84, *p* < 0.05; *Interaction*: F_(33, 572)_ = 7.855, *p* < 0.05; NEPD: *Dose*: F_(3, 44)_ = 43.63, *p* < 0.05; *Time*: F_(11, 484)_ = 15.03, *p* < 0.05; *Interaction*: F_(33, 484)_ = 6.783, *p* < 0.05; NEP: *Dose*: F_(3, 51)_ = 78.22, *p* < 0.05; *Time*: F_(11, 561)_ = 116.0, *p* < 0.05; *Interaction*: F_(33, 561)_ = 29.81, *p* < 0.05; and 4-MeAP: *Dose*: F_(3, 51)_ = 78.22, *p* < 0.05; *Time*: F_(11, 561)_ = 116.0, *p* < 0.05; *Interaction*: F_(33, 561)_ = 29.81, *p* < 0.05. HLA time courses revealed a rapid onset effect (5–10 min) for all the compounds after 10 and 30 mg/kg injections. At the lowest and medium doses tested, the increase in locomotor activity ended before 60 min, except for NEPD and NEP, whose effect at 10 mg/kg seems to persist more than 1 hour. It is important to note the decreasing slope of NEPD and NEP during the first 20 min at a dose of 30 mg/kg, probably due to the observed increase in stereotypes as mentioned before.

When analyzing the HLA induced by all the compounds at the medium dose tested (10 mg/kg), as well as the hyperlocomotion induced by cocaine in an independent experiment at the same dose (distance traveled: cocaine 10 mg/kg = 16,018 ± 1,118 cm, *N* = 15), one-way ANOVA yielded a significant effect of the variable *Drug* (F_(6, 83)_ = 45.29, *p* < 0.05). Subsequent post hoc Tukey’s test revealed the following rank order of effectiveness: NEP = NEPD > pentylone ≥ pentedrone = cocaine = 4-MeAP ≥ 4-MPD.

### Effect on Conditioned Place Preference

**FIGURE 6:**
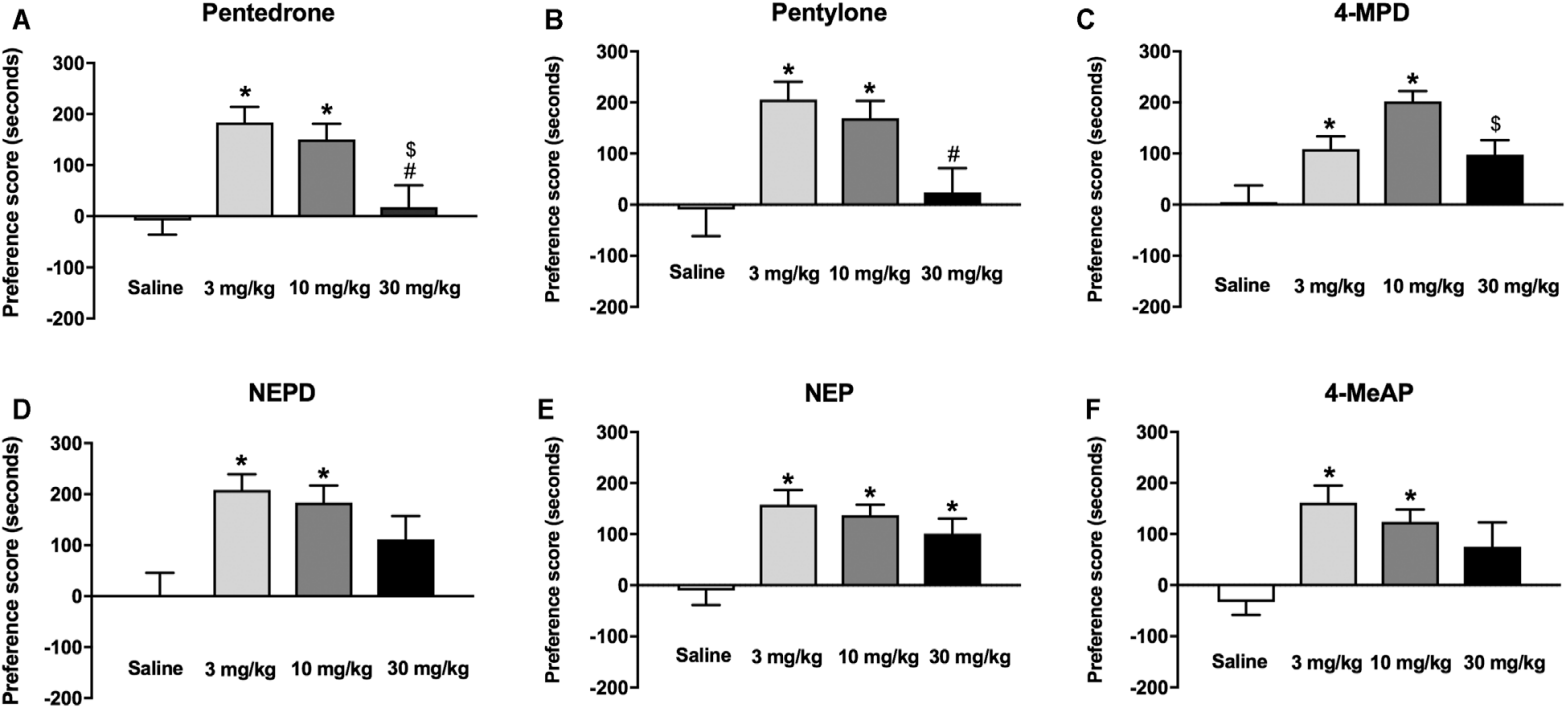
Effects of pentedrone **(A)**, pentylone **(B)**, 4-MPD **(C)**, NEPD **(D)**, NEP **(E)**, and 4-MeAP **(F)** on the CPP test in mice. Bars represent the mean ± SEM of the preference score (difference between the time spent in the drug-paired compartment on the test day and the preconditioning day). *Tukey’s* multiple-comparisons test; ^*^ *p* < 0.05 vs the saline group; ^#^ *p* < 0.05 vs the 3 mg/kg dose group; ^$^ *p* < 0.05 vs the 10 mg/kg dose group (*N* = 12–14/group).

The rewarding effects of pentedrone, pentylone, NEPD, NEP, 4-MeAP, and 4-MPD were studied using the CPP paradigm. No statistical differences were found between percentages of time spent in each compartment (around 50% each) during the preconditioning phase of all CPP experiments, indicating a lack of preference for either compartment. Three animals were withdrawn from the experiments due to an initial preference for one of the compartments (>70% of the total session time). On the test day, one-way ANOVA yielded a significant effect of *Dose* for all the synthetic cathinones tested (pentedrone: F_(3, 44)_ = 8.063, *p* < 0.05; pentylone: F_(3, 44)_ = 6.028, *p* < 0.05; NEPD: F_(3, 44)_ = 5.626, *p* < 0.05; NEP: F_(3, 51)_ = 7.632, *p* < 0.05; 4-MPD: F_(3, 51)_ = 8.798, *p* < 0.05; 4-MeAP: F_(3, 51)_ = 5.866, *p* < 0.05). As shown in Figures 6A–F, all compounds share a similar conditioning profile, inducing a significant increase in the preference score at 3 and 10 mg/kg compared to their corresponding saline-treated group. However, only NEP-treated mice showed a significant increase of the rewarding effects at the highest dose tested (30 mg/kg). Additionally, while conditioning with N-methylamino derivatives pentedrone, pentylone, and 4-MPD at 3 and/or 10 mg/kg induced a significant increase of the preference score compared to the highest dose tested (30 mg/kg), no significant differences were found among doses for N-ethylamino derivatives NEPD, NEP, and 4-MeAP.

### Effect on IEG Expression

In order to evaluate the ability of different ring-substituted *α*-aminovalerophenone derivatives to induce an overexpression of some IEGs, mRNA levels of *Arc* and *c-fos* were determined in both the ventral striatum and the dorsal striatum 30 and 120 min after a single acute administration (10 mg/kg, i.p.) of pentedrone, pentylone, NEPD, NEP, 4-MeAP, or 4-MPD in mice. In addition, the expression of mRNA levels of *bdnf* was also assessed in the mPFC 120 min after administration.

#### Arc mRNA Expression

**FIGURE 7:**
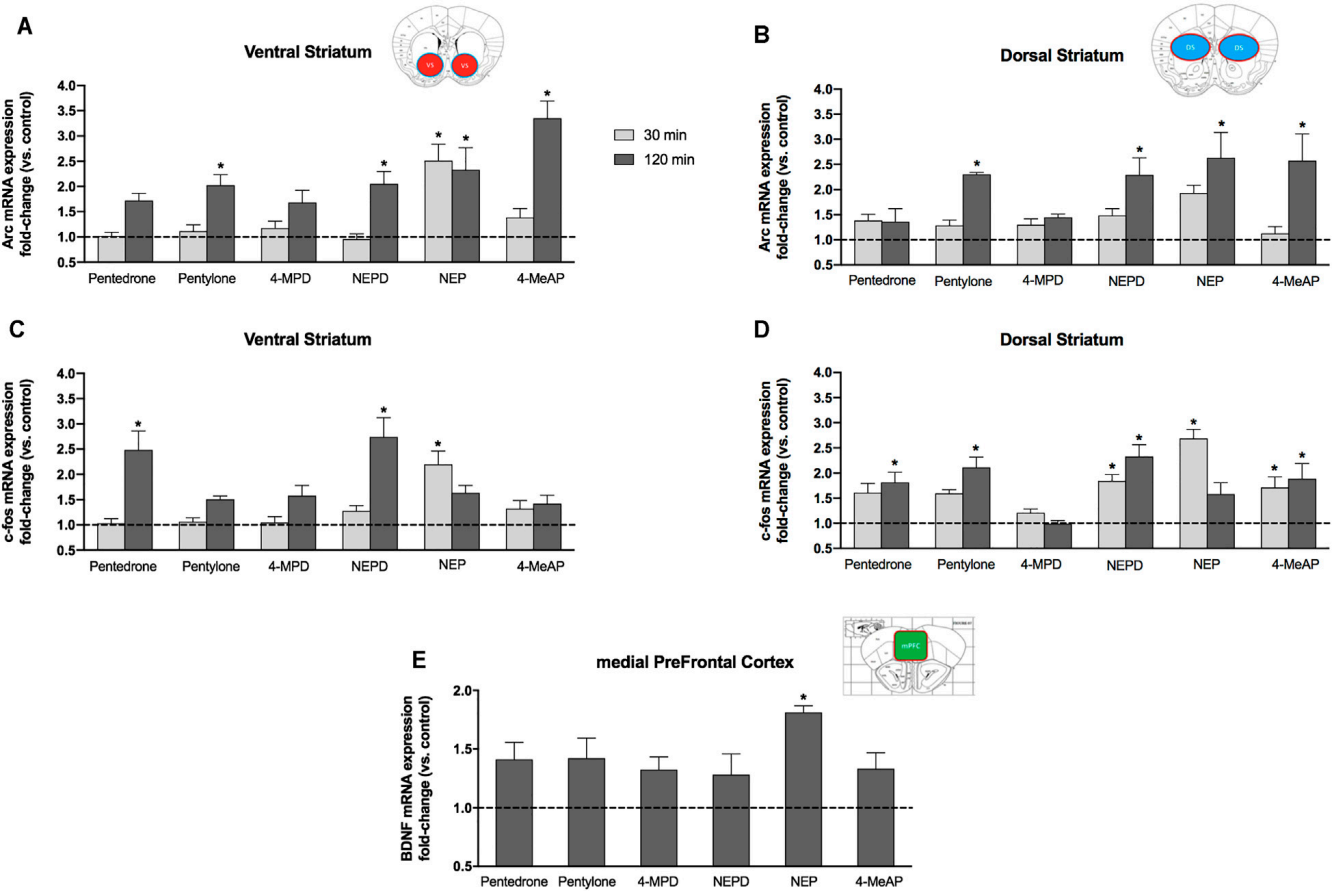
Effects on *Arc* **(A,B)** and *c-fos* **(C,D)** mRNA expressions induced by a single acute dose (10 mg/kg, i.p.) of pentedrone, pentylone, 4-MPD, NEPD, NEP, and 4-MeAP in the mouse VS **(A,C)** and DS **(B,D)** at 30 and 120 min after administration. **(E)** Effects on *bdnf* mRNA expression induced by a single acute dose (10 mg/kg, i.p.) of pentedrone, pentylone, 4-MPD, NEPD, NEP, and 4-MeAP in the mouse mPFC at 120 min after administration. Data are expressed as the mean ± SEM of fold changes in mRNA levels vs the corresponding saline group (*N* = 5–6 mice/group). Samples were tested in duplicate. *Dunnett’s* multiple-comparisons test; *p < 0.05 vs the corresponding saline group (30 or 120 min).

Two-way ANOVA of the results revealed that not only *Arc* gene expression in the VS was significantly affected by the variables *Drug* (F_(6.62)_ = 11.42, *p* < 0.05) and *Time* (F _(1.62)_ = 35.90, *p* < 0.05) but also a significant interaction *Drug x Time* was obtained (F_(6.62)_ = 5.125, *p* < 0.05). Regarding the DS, two-way ANOVA of the results yielded a significant effect of the variable *Drug* on *Arc* gene expression in the DS (F_(6.64)_ = 5.518, *p* < 0.05), the variable *Time* (F_(1.64)_ = 18.36, *p* < 0.05), and the interaction of both variables (F_(6.64)_ = 2.408, *p* < 0.05). Acute injection of pentylone, NEPD, NEP, and 4-MeAP induced a significant increase of *Arc* gene expression in the VS and DS 120 min after administration (Figures 7A,B). Furthermore, NEP was also able to induce a similar increase of mRNA levels in the VS 30 min after injection. No significant increases of *Arc* gene expression in the DS were induced by any compound tested 30 min after injection.

#### c-fos mRNA Expression

The expression of the mRNA levels of the *c-fos* gene in the VS was found to be significantly affected by the variable *Drug* (F_(6.65)_ = 7.980, *p* < 0.05), the variable *Time* (F_(1.65)_ = 22.95, *p* < 0.05), and the *Drug x Time* interaction (F_(6.65)_ = 7.732, *p* < 0.05). Two-way ANOVA also revealed a significant effect of the variable *Drug* on the *c-fos* gene in the DS (F_(6.67)_ = 12.59, *p* < 0.05) and the *Drug x Time* interaction (F_(6.67)_ = 4.888, *p* < 0.05), but not the variable *Time* (F_(1.67)_ = 0.007, *p* > 0.05). Pentedrone and NEPD induced a significant increase of *c-fos* expression in the VS 120 min after administration. Moreover, 30 min after injection, only NEP-treated mice showed a significant overexpression of the *c-fos* gene (Figure 7C). Regarding the DS and *c-fos* mRNA expression, pentylone, pentedrone, NEPD, and 4-MeAP administration increased *c-fos* expression 120 min post injection. However, only NEPD and 4-MeAP also induced a significant overexpression of *c-fos* levels 30 min after injection. Similar to the VS, NEP only increased *c-fos* mRNA levels in the DS 30 min after administration (Figure 7D).

#### bdnf mRNA Expression

One-way ANOVA of the results revealed a significant effect of the variable *Drug* (F_(6.33)_ = 3.525, *p* < 0.01). Although a tendency to increase *bdnf* expression was observed after an acute administration of all compounds tested, only NEP-treated mice showed a significant overexpression of *bdnf* mRNA levels (Figure 7E).

## Discussion

First, we focused on the ability of these novel synthetic cathinones to act at the DAT and SERT. Our results demonstrated that all the synthetic cathinones tested are equally or even more potent than cocaine in inhibiting the DAT. Particularly, the potency at inhibiting DA uptake increased between two- and fourfold in N-ethyl–substituted cathinones in comparison with N-methyl–substituted cathinones. This is in accordance with previous studies published by our research group in which we observed decreased DAT IC_50_ values when increasing the length of the amino group of no ring-substituted *α*-aminovalerophenone derivatives (Duart-Castells et al., 2021). In parallel, when looking at SERT inhibition, all the ring-substituted cathinones tested showed higher potency at inhibiting 5-HT uptake than their non-substituted analogs. These results correlate with previous findings in which the addition of a 3,4-methylenedioxy-group or *para*-substitutions on the phenyl ring of methcathinone generally shifts selectivity toward the SERT (Cozzi et al., 1999; Bonano et al., 2015). However, it must be pointed out that this SAR does not apply to other synthetic cathinones such as MDPV and *α*-PVP and other *para*-substituted pyrrolidino-valerophenone derivatives (Eshleman et al., 2019). Further studies are needed in order to investigate the interaction as well as the SAR of these compounds with other monoaminergic transporters and receptors.

Interestingly, none of the synthetic cathinones tested elicited release at the DAT, but most of them elicited at least partial release at the SERT. These results emphasized the hybrid and partial transporter activity of some synthetic cathinones, as previously described for pentylone (Saha et al., 2019). More precisely, besides NEP, all tested synthetic cathinones act as substrates at the SERT but as inhibitors at the DAT.

Previous studies have reported a correlation between DAT selectivity relative to the SERT (i.e., DAT/SERT ratio) and abuse liability (Negus and Banks, 2017; Simmler and Liechti, 2017). In fact, the MDMA-like subjective effects of pentedrone and self-administration of pentylone are suggested to be related to their limited serotonergic potency relative to their dopaminergic efficacy (Dolan et al., 2018; Gatch et al., 2020). In our study, all the compounds tested showed higher DAT/SERT ratios than cocaine, indicating a high abuse potential. In particular, N-ethyl– and/or non-substituted synthetic cathinones tested possess higher DAT selectivity than their corresponding N-methyl– and/or ring-substituted analogs, respectively.

During the last decade, several studies have demonstrated that synthetic cathinones are able to increase locomotor activity and therefore induce a strong psychostimulant effect (Marusich et al., 2014; Giannotti et al., 2017; Hwang et al., 2017; Javadi-Paydar et al., 2018; Wojcieszak et al., 2018). It is particularly interesting to note the different locomotor activity profile of pentedrone, pentylone, and their N-ethyl analogs. While pentedrone and NEPD induced a ceiling effect at the highest dose tested, pentylone produced a typical dose-increase response. In fact, time- and dose-dependent stimulation on locomotor activity was previously reported for pentedrone (2.5–25 mg/kg) and pentylone (10–100 mg/kg) (Gatch et al., 2015). On the contrary, NEP showed an inverted U-shaped dose-response curve due to the emergence of focused stereotypes such as sniffing and head bobbing at the higher dose tested (Baumann et al., 2018). This inverted U-shaped dose-response curve of the locomotor activity is not unique of NEP, but a common effect of stimulants, since other authors have observed the same effect not only for NEP (Gatch et al., 2019; Li et al., 2019) but also for other novel synthetic cathinones, such as eutylone (Glatfelter et al., 2021).

To our knowledge, there is no information about the psychostimulant effect induced by the *para*-methyl–substituted cathinones tested in this study, 4-MPD and 4-MeAP. Our results demonstrated that 4-MPD was the less efficacious compound eliciting hyperlocomotion and 4-MeAP was equally effective as cocaine at the same dose. Furthermore, NEP and NEPD were more efficacious at a dose of 10 mg/kg than their N-methyl analogs, pentylone and pentedrone, which correlates with their higher potency and selectivity inhibiting the DAT. However, some discrepancies were found when comparing the *in vitro* and *in vivo* results of 4-MPD and 4-MeAP. Although 4-MPD and 4-MeAP have similar potency and selectivity inhibiting the DAT when compared with pentylone and NEP, respectively, they are significantly less efficacious eliciting hyperlocomotion than their 3,4-methylenedioxy analogs. Similar discrepancies were also recently reported for other novel synthetic cathinones, such as α-piperidinevalerophenone (α-PVP) and dibutylone, in previous studies (Duart-Castells et al., 2021; Glatfelter et al., 2021). Based on these observations, we can consider that the pharmacokinetics of these *para*-methyl–substituted cathinones could affect brain levels of the drugs. For example, Grecco et al. (2017) demonstrated that common structural modifications of synthetic cathinones might yield different plasma levels, CNS, and plasma elimination rates as well as affecting brain penetration of the compounds.

The results reported in the present study demonstrated for the first time that NEP, 4-MPD, and 4-MeAP are able to induce reward, similar to other classical psychostimulants such as cocaine (López-Arnau et al., 2017). All the compounds induced significant rewarding effects at the lowest and medium doses tested (3 and 10 mg/kg). Previous studies have already demonstrated the reinforcing properties of pentylone in rats using a self-administration paradigm (Dolan et al., 2018). Surprisingly, although NEP showed an inverted U-shaped dose-response curve in the HLA, it was the only synthetic cathinone able to produce rewarding effects at the highest dose tested. Additionally, in release assays, this synthetic cathinone was the only one in the group not inducing mild to moderate ^3^[H]5-HT release, as it has already been previously described (Costa et al., 2019).

To our knowledge, little is known about the induction, in specific brain areas (DS, VS, and mPFC), of different IEGs related to addiction after an acute exposure to recently emerged novel synthetic cathinones, such as pentylone, pentedrone, NEP, NEPD, 4-MeAP, and 4-MPD. Our results showed an overexpression of *Arc* mRNA levels in both the DS and the VS 2 h after administration of NEPD, NEP, 4-MeAP, and pentylone. Therefore, the observed up-regulation of this IEG may induce prolonged neuroadaptations and cause alterations in the cell structure affecting the morphology of neurons (Fosnaugh et al., 1995; Fumagalli et al., 2006). Regarding other synthetic cathinones, it has been demonstrated that acute MDPV and α-PVP administration induces an up-regulation of *Arc* mRNA levels in the striatum (Giannotti et al., 2017; Wojcieszak et al., 2019). Specifically, the acute injection of MDPV increased *Arc* mRNA levels 30 min after administration and persisted 2 h later. However, α-PVP injection did not alter its expression at any time point tested. According to Giannotti et al. (2017), the difference observed in *Arc* expression between both substances might be correlated with a greater potency to inhibit the DAT (Marusich et al., 2014) and higher psychostimulant effect induced by MDPV in comparison with α-PVP. This is partially in accordance with our *in vitro* and *in vivo* results. On the one hand, all the N-ethyl–substituted analogs (NEPD, NEP, and 4-MeAP) were the more potent compounds inhibiting DA uptake. On the other hand, NEPD, NEP, and pentylone were the most effective compounds in inducing hyperlocomotion.

*c-fos* is a neuronal activity marker, its expression indicates a response of adaptation, memory formation, and neuroplasticity, and its expression increases after extracellular signals such as ions, neurotransmitters, and growth factor drugs (Kovács, 2008; Gallo et al., 2018). All the synthetic cathinones tested in this study induced a significant increase in *c-fos* expression in the DS 30 and/or 120 min post administration, except for 4-MPD. This observation is in accordance with our *in vivo* results in which we demonstrated that 4-MPD was the least effective compound in inducing an increase in the locomotion. Regarding the VS, and in contrast to the DS, the acute administration of only NEPD, NEP, and pentedrone was able to increase significantly *c-fos* expression when compared to the corresponding saline group at 30 or 120 min after injection. Altogether, this indicates that these structurally related synthetic cathinones may affect IEG expression but with a different gene expression profile. However, it must be pointed out that NEPD and NEP were the only substances tested able to up-regulate *Arc* and *c-fos* levels in both the DS and the VS, which correlates with their higher potency and selectivity toward the DAT. In fact, the increased synaptic levels of dopamine could activate D1 dopamine receptors, leading to increased *Arc/Arg3.1* and *c-fos* mRNA levels, as previously demonstrated (Yoshida et al., 1995; Fumagalli et al., 2006).

In the present study, the observed increase in *bdnf* expression after NEP administration may start causing changes in neuronal signaling and synaptic strength (Zhang et al., 2002; Le Foll et al., 2005; Rodriguez-Espinosa and Fernandez-Espejo, 2015). Moreover, the up-regulation of *bdnf* may explain the more sustained *Arc* levels over time induced by NEP and other synthetic cathinones such as MDPV (Giannotti et al., 2017; Duart-Castells et al., 2019) since *bdnf* is able to enhance the synthesis of Arc protein and up-regulate *Arc* mRNA levels (Yin et al., 2002; Ying et al., 2002). Moreover, NEP was the most potent compound acting as an SERT blocker, which correlates with the link that exists between *bdnf* and 5-HT (Homberg et al., 2014; Popova et al., 2017), as well as selective 5-HT reuptake inhibitors (SSRIs) used in antidepressant treatment and an enhanced *bdnf* gene expression (Martinowich and Lu, 2008; Arosio et al., 2021; Casarotto et al., 2021).

In summary, the present study demonstrates the increased DAT inhibition potency of N-ethyl vs N-methyl synthetic cathinones as well as their increased selectivity toward the SERT in *para*-methyl– and 3,4-methylenedioxy–substituted compounds. Moreover, we must highlight the “hybrid” mechanism of action of most of the novel synthetic cathinones tested in this study. Finally, the *in vitro* results partially correlate not only with the psychostimulant and rewarding effects induced by the novel synthetic cathinones tested in this study but also with the induction of some IEGs related to addiction.

## Data Availability

The original contributions presented in the study are included in the article/Supplementary Material, and further inquiries can be directed to the corresponding authors.
