# Supplementary material for: Structure–Activity Relationship of Novel Second-Generation Synthetic Cathinones: Mechanism of Action, Locomotion, Reward, and Immediate-Early Genes
Source: Front Pharmacol. 2021 Oct 26;12:749429. doi: 10.3389/fphar.2021.749429 (PMC8576102; doi:10.3389/fphar.2021.749429)
Supplement: Supplementary file 1 [file Table1.DOCX]

|  |  | **Release assays** | | |
| --- | --- | --- | --- | --- |
|  |  | **Statistical results (F and p value)** | | |
| **Compound** | **Variables** | **hDAT [IC_50_]** | **hDAT [10 µM]** | **hSERT [IC_50_]** |
| **Pentedrone / Pentedrone + Mon.** | Time | F_(1.391,13.32)_ = 38.31 ; p<0.001 | F_(2.272,22.4)_ = 48.66 ; p<0.001 | F_(2.815,28.15)_ = 39.68 ; p<0.001 |
|  | Drug | F_(1,10)_ = 6.067 ; p<0.05 | F_(1,10)_ = 0.5027 ; p>0.05 | F_(1,10)_ = 29.17 ; p<0.001 |
|  | Time x Drug | F_(7,67)_ = 6.756 ; p<0.001 | F_(7,69)_ = 13.35 ; p<0.001 | F_(7,70)_ = 16.98 ; p<0.001 |
| **Pentylone /**  **Pentylone + Mon.** | Time | F_(1.700,33.52)_ = 18.64 ; p<0.001 | F_(1.49,32.79)_ = 2.581 ; p>0.05 | F_(2.301,26.30)_ = 150.7 ; p<0.001 |
|  | Drug | F_(1,20)_ = 1.871 ; p>0.05 | F_(1,22)_ = 0.061 ; p>0.05 | F_(1,12)_ = 37.83 ; p<0.001 |
|  | Time x Drug | F_(7,138)_ = 0.9749 ; p>0.05 | F_(7,154)_ = 0.3584 ; p>0.05 | F_(7,80)_ = 47.46 ; p<0.001 |
| **4-MPD /**  **4-MPD + Mon.** | Time | F_(2.644,37.02)_ = 2.606 ; p>0.05 | F_(1.204,12.04)_ = 0.2367 ; p>0.05 | F_(2.583,25.83)_ = 120.1 ; p<0.001 |
|  | Drug | F_(1,14)_ = 0.00996 ; p>0.05 | F_(1,10)_ = 0.1006 ; p>0.05 | F_(1,10)_ = 91,09 ; p<0.001 |
|  | Time x Drug | F_(7,58)_ = 1.808 ; p>0.05 | F_(7,70)_ = 1.548 ; p>0.05 | F_(7,70)_ = 48,71 ; p<0.001 |
| **NEPD /**  **NEPD + Mon.** | Time | F_(1.584,15.39)_ = 21.84 ; p<0.001 | F_(2.942,29.42)_ = 38.86 ; p<0.001 | F_(2.321,21.89)_ = 85.33 ; p<0.001 |
|  | Drug | F_(1,10)_ = 5.054 ; p<0.05 | F_(1,10)_ = 0.7310 ; p>0.05 | F_(1,10)_ = 127.2 ; p<0.001 |
|  | Time x Drug | F_(7,68)_ = 3.192 ; p<0.01 | F_(7,70)_ = 4.888 ; p<0.001 | F_(7,66)_ = 35.03 ; p<0.001 |
| **NEP /**  **NEP + Mon.** | Time | F_(1.854,25.95)_= 30.96 ; p<0.001 | F_(1.467,14.67)_ = 9.711 ; p<0.01 | F_(2.238 ; 30.38)_ = 60.30 ; p<0.001 |
|  | Drug | F_(1,14)_ = 2.211 ; p>0.05 | F_(1,10)_ = 0,3262 ; p>0.05 | F_(1,14)_ = 16.39 ; p<0.01 |
|  | Time x Drug | F_(7,98)_= 2.013 ; p>0.05 | F_(7,70)_ = 0,1425 ; p>0.05 | F_(7,95)_ = 19.07 ; p<0.001 |
| **4-MeAP /**  **4-MeAP + Mon.** | Time | F_(3.894,54.52)_ = 26.88 ; p<0.001 | F_(2.494,24.94)_ = 13.02 ; p<0.001 | F_(1.925;19.25)_ = 142.7 ; p<0.001 |
|  | Drug | F_(1,14)_= 0.00002335 ; p>0.05 | F_(1,10)_ = 4.275 ; p>0.05 | F_(1,10)_ = 123.6 ; p<0.001 |
|  | Time x Drug | F_(7,98)_= 0.2309 ; p>0.05 | F_(7,70)_ = 3.426 ; p<0.01 | F_(7,70)_ = 64,69 ; p<0.001 |
| **Paroxetineª /**  **Paroxetineª + Mon.** | Time | N.A. | N.A. | F_(3.398,57.76)_ = 211.6 ; p<0.001 |
|  | Drug | N.A. | N.A. | F_(1,18)_ = 7.128 ; p<0.05 |
|  | Time x Drug | N.A. | N.A. | F_(7,119)_ = 8.773 ; p<0.001 |
| **PCAª /**  **PCAª + Mon.** | Time | N.A. | N.A. | F_(2.1699,43.18)_ = 431 ; p<0.001 |
|  | Drug | N.A. | N.A. | F_(1,112)_ = 96.27 ; p<0.001 |
|  | Time x Drug | N.A. | N.A. | F_(7,112)_ = 121.5 ; p<0.001 |
| **GBRª /**  **GBRª + Mon.** | Time | F_(1.675,36.62)_ = 24.21 ; p<0.001 | N.A. | N.A. |
|  | Drug | F_(1,22)_ = 0.0002409 ; p>0.05 | N.A. | N.A. |
|  | Time x Drug | F_(7,153)_ = 6.062 ; p<0.001 | N.A. | N.A. |
| **Amphetamineª /**  **Amphetamineª + Mon.** | Time | F_(3.053,75.45)_ = 392.8 ; p<0.001 | N.A. | N.A. |
|  | Drug | F_(1,25)_ = 77.90 ; p<0.001 | N.A. | N.A. |
|  | Time x Drug | F_(7,173)_ = 32.32 ; p<0.001 | N.A. | N.A. |

**Table SM1.** Statistical results (mixed-effects model) of the transport-mediated release assays**.**

N.A., not assessed; ª Control compounds.
