# Supplementary figures and images for: Structure–Activity Relationship of Novel Second-Generation Synthetic Cathinones: Mechanism of Action, Locomotion, Reward, and Immediate-Early Genes
Source: Front Pharmacol. 2021 Oct 26;12:749429. doi: 10.3389/fphar.2021.749429 (PMC8576102; doi:10.3389/fphar.2021.749429)

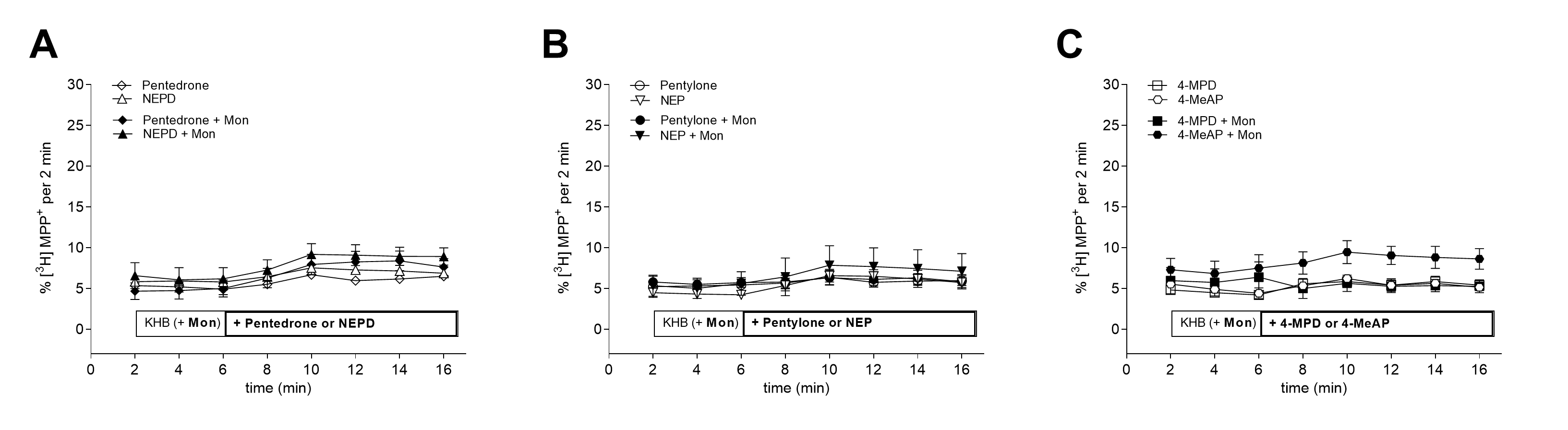

Supplement: Supplementary file 2 [file Image2.TIF]

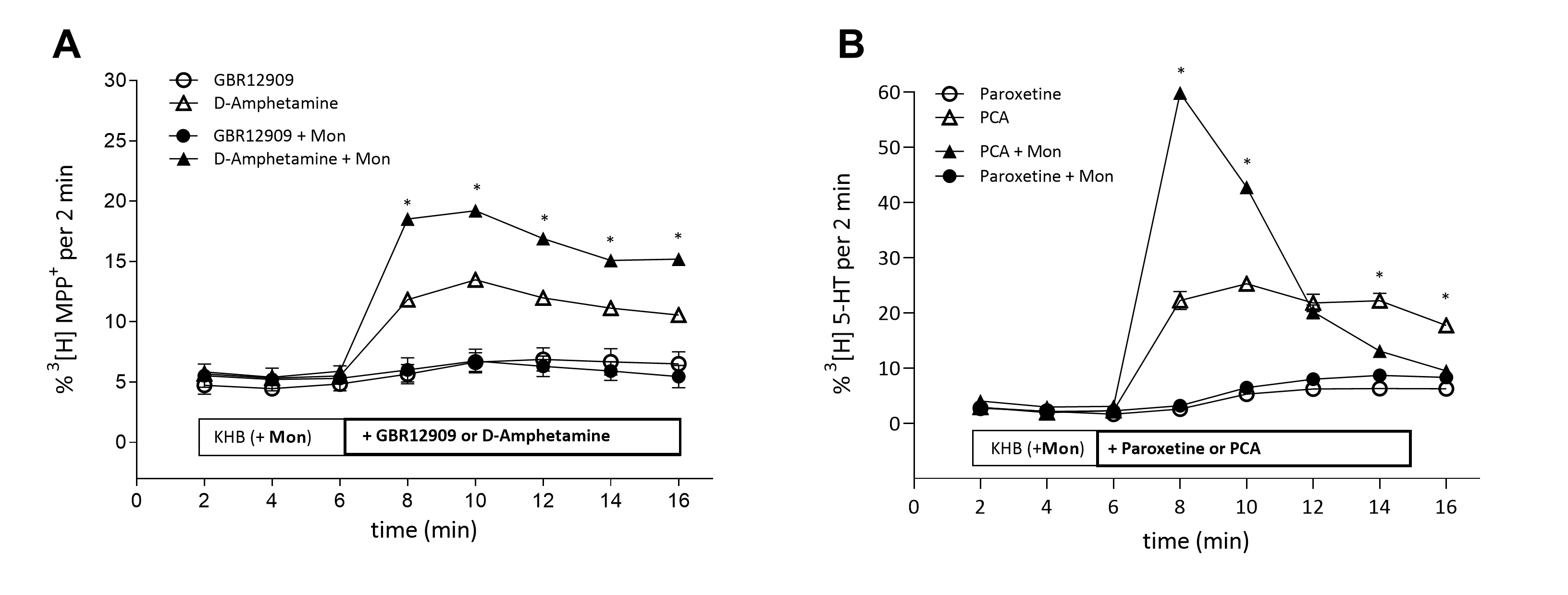

Supplement: Supplementary file 3 [file Image1.TIF]
