# Supplementary material for: Structure–Activity Relationship of Novel Second-Generation Synthetic Cathinones: Mechanism of Action, Locomotion, Reward, and Immediate-Early Genes
Source: Front Pharmacol. 2021 Oct 26;12:749429. doi: 10.3389/fphar.2021.749429 (PMC8576102; doi:10.3389/fphar.2021.749429)
Supplement: Supplementary file 4 [file DataSheet1.docx]

Supplementary Material

# Supplementary Data

**Synthesis and chemical characterization**

The synthesis and characterization of the synthetic cathinones was carried out through three steps, following the procedure formerly described by Meltzer et al., 2006 and Duart-Castells et al., 2021, with minor modifications. Chemical purity and identification of the obtained compound was also assessed by TLC, ^1^H NMR, ^13^C NMR, IR and MS. The corresponding ring-substituted nitrile compound was subjected to reaction with n-Butylmagnesium chloride (n-BuMgCl), followed by acidic hydrolysis, to yield the intermediate ketone (Step 1). The reaction was performed in anhydrous conditions. After 3 h at room temperature, the reaction was completed. Then, the reaction mixture was cooled, and a 4% solution of sulfuric acid (H_2_SO_4_) (4%) was added drop by drop. Phases were separated, the aqueous layer extracted with diethyl ether (Et_2_O) and the organic phases were combined and dried with magnesium sulfate (MgSO_4_), filtered and reduced in vacuo to an oil. The α-bromination (Step 2) was achieved by the addition of bromine (Br_2_) in a dropwise manner to a solution of the intermediate ketone in dichloromethane (CH_2_Cl_2_) in presence of acetic acid (AcOH) in catalytic amounts (Step 2). After 1.5 h the reaction was completed and the excess of bromine neutralized with an aqueous solution of sodium thiosulfate (Na_2_S_2_O_3_). The organic layer was separated, dried (MgSO_4_), filtrated and reduced in vacuo to an oil. The α‑bromoketone was dissolved in ethanol (EtOH), and the amine was added all at once (Step 3). The reaction was heated when necessary. After 2-24 h the reaction was completed. Then, the reaction mixture was extracted with hydrochloric acid (HCl) 1 N in water and then back-extracted into Et_2_O by basification to pH 10 with sodium hydroxide (NaOH) 1 N. The organic layer was dried with MgSO_4_, filtered and reduced in vacuo to an oil. Each product was dissolved in EtOH and a mixture of Et_2_O with HCl (3 M) in cyclopentyl methyl ether (CPME) was added in a dropwise manner in order to obtain the hydrochloride salt. Solids were collected by filtration. The identification of the cathinone was assessed as previously described yielding the following results:

**α-methylaminovalerophenone (Pentedrone) hydrochloride**:

^1^H-NMR (400 MHz, Chloroform-*d*) 𝛿: 10.55 (s, 1H), 9.16 (s, 1H), 7.96 (dd, *J* = 8.4, 1.2 Hz, 2H), 7.70 – 7.64 (m, 1H), 7.53 (t, *J* = 7.8 Hz, 2H), 4.94 (s, 1H), 2.81 (s, 3H), 2.30 – 2.16 (m, 1H), 2.16 – 2.02 (m, 1H), 1.61 – 1.46 (m, 1H), 1.45 – 1.29 (m, 1H), 0.89 (t, *J* = 7.3 Hz, 3H)

^13^C NMR (100 MHz, Chloroform-*d*): δ 194.68 , 135.03 , 134.03 , 129.42 , 128.88 , 63.29 , 32.61 , 32.45 , 18.24 , 14.02 .

IR (KBr), v max: 2933, 2774, 1680, 1464, 1346, 1245, 1002, 708 cm^-1^.

m/z: 192 (M+1)

**α-ethylaminovalerophenone (NEPD) hydrochloride**:

^1^H-NMR (400 MHz, Chloroform-d) 𝛿(ppm): 7.99 – 7.98 (d, J = 7.4 Hz, 2H), 7.72 – 7.68 (t, J = 7.4Hz, 1H), 7.58 – 7.54 (t, J = 7.6 Hz, 2H), 4.97 (s, 1H), 3.24 (s, 1H), 3.04 (s, 1H), 2.33 –2.29 (d, J = 12.4 Hz, 1H), 2.21 – 2.14 (m, 1H), 1.56 – 1.53 (t, J = 7.2 Hz, 3H), 1.48 – 1.32 (m, 2H), 0.90 – 0.86 (t, J = 7.2 Hz, 3H).

^13^C NMR (100 MHz, DMSO-*d*_6_) δ 196.77 , 135.21 , 134.46 , 129.63 , 129.18 , 60.87 , 41.59 , 32.20 , 17.61 , 14.10 , 11.57 .

IR (KBr) 𝜈max (cm-1): 3428, 2960, 1687, 1449, 1237, 1002, 709 cm^-1^.

m/z : 206 (M+1).

**3’,4’-methylendioxy-α-methylaminovalerophenone (Pentylone) hydrochloride**:

^1^H-NMR (400 MHz, DMSO-d6) 𝛿: 9.56 (s, 1H), 9.11 (s, 1H), 7.72 (dd, J = 8.2, 1.8Hz, 1H), 7.54 (d, J = 1.8 Hz, 1H), 7.13 (d, J = 8.2 Hz, 1H), 6.19 (s, 2H), 5.16– 5.13 (t, J = 7.2 Hz, 1H), 2.53 (t, , J = 5.0 Hz, 3H), 1.89 – 1.79 (m, 2H), 1.33 –1.24 (m, 1H), 1.13 – 1.06 (m, 1H), 0.81 – 0.78 (t, J = 7.2 Hz, 3H) ppm.

^13^C-NMR (100 MHz, DMSO-dd) 𝛿: 194.1, 152.8 , 148.2, 128.6, 125.8, 108.5, 107.8, 102.5, 61.6, 31.9, 31.3, 17.1, 13.7 ppm.

IR (KBr), v max: 3421, 2961, 2763, 1673, 1456, 1259, 1105, 1033, 927 cm^-1^.

m/z: 236 (M+1)

**3’,4’-methylendioxy-α-ethylaminovalerophenone (NEP) hydrochloride**:

^1^H-NMR (400 MHz, Chloroform-*d*) δ (ppm): 11.26 (s, 1H), 7.91 (s, 1H), 7.59 (dd, J = 8.2, 1.7 Hz, 1H), 7.45 (d, J = 1.7 Hz, 1H), 6.93 (d, J = 8.2 Hz, 1H), 6.10 (s, 2H), 4.91 – 4.84 (m, 1H), 3.27 – 3.16 (m, 1H), 3.00 – 2.93 (m, 1H), 2.35 – 2.22 (m, 1H), 2.21 – 2.08 (m, 1H,), 1.52 (t, J = 7.3 Hz, 3H), 1.48 – 1.38 (m, 1H), 1.37 – 1.24 (m, 1H), 0.89 (t, J = 7.3 Hz, 3H).

13C-NMR (100 MHz, Chloroform-*d*) δ (ppm): 193.06, 153.67, 149.00, 128.98, 126.04, 108.66, 108.34, 102.52, 61.39, 42.94, 33.31, 18.49, 14.03, 11.96.
IR (KBr) νmax (cm-1): 3427, 2961, 1673, 1449, 1354, 1257, 1035 cm^-1^.
MS *m/z*: 250 (M+1)

**4’-methyl-α-methylaminovalerophenone (4-MPD) hydrochloride**:

^1^H-NMR (400 MHz, DMSO-*d_6_*) 𝛿: 9.51 (s, 1H), 9.13 (s, 1H), 7.95 (d, *J* = 8.3 Hz, 2H,), 7.43 – 7.41 (d, *J* = 8.3 Hz, 2H), 5.19 (t, *J* = 5.5 Hz, 1H), 2.57 – 2.55 (t, 3H), 2.41 (s, 3H,), 1.91 – 1.78 (m, 2H), 1.33 – 1.26 (m, 1H), 1.11 – 1.04 (m, 1H), 0.80 – 0.76 (t, *J* = 7.3 Hz, 3H) ppm.

^13^C-NMR (100 MHz, DMSO-*d_d_*) 𝛿: 195.6, 145.6, 131.4, 129.7, 128.8, 61.8, 31.7, 31.4, 21.3, 17.1, 13.6 ppm.

IR (KBr) 𝜈max (cm-1): 2955, 2773, 1687, 1464, 1233, 1006, 769 cm^-1^.

m/z: 206 (M^+1^)

**4’-methyl-α-ethylaminovalerophenone (4-MeAP) hydrochloride**:

^1^H-NMR (400 MHz, DMSO-*d_6_*) 𝛿: 9.49 (s, 1H,), 9.03 (s, 1H), 7.98 (d, *J* = 8.3 Hz, 2H), 7.42 (d, *J* = 8.3 Hz, 2H), 5.21 (t, *J* = 5.6 Hz, 1H), 3.05 – 2.97 (m, 1H), 2.92 – 2.85 (m, 1H), 2.41 (s, 3H), 1.95 – 1.80 (m, 2H), 1.32 (dd, *J* = 9.1, 4.1 Hz, 1H),1.25 (t, *J* = 7.3 Hz, 3H), 1.11 – 1.02 (m, 1H), 0.79 – 0.76 (t, *J* = 7.3 Hz, 3H) ppm.

^13^C-NMR (100 MHz, DMSO-*d_d_*) 𝛿: 195.8, 145.6, 131.5, 129.8, 128.9, 60.3, 41.1, 31.9, 21.3, 17.2, 13.7 and 11.1 ppm.

IR (KBr) 𝜈max (cm-1): 2940, 2739, 1688, 1432, 1232, 1006, 771 cm^-1^.

m/z: 220 (M+1)

**Supplementary Figure 1.** Effects of paroxetine, p-chloroamphetamine (PCA), GBR12909 and D-amphetamine on transport-mediated release of preloaded radiolabelled substrate from HEK293 cells stably expressing (A) SERT or (B) DAT. As indicated, after 6 min of basal release (either in the presence of KHB or KHB with monensin 10µM), negative controls for each cell line were added at a concentration close to their predetermined IC50 value for each transporter (paroxetine (0.05µM), GBR12909 (0.5µM)), whereas positive controls were added at a concentration that evokes high release on each transporter (PCA (10µM), D-amphetamine (10µM)). The effects of each compound in the presence of KHB or KHB with monensin 10μM are shown as empty or filled symbols, respectively. Data are mean ± SEM for n=4-7 experiments performed in duplicate. Data was statistically analyzed using with a mixed-effects model, employing Šidák’s correction for multiple comparisons. This statistical analysis explored possible significant differences between KHB + compound and KHB with monensin 10µM + compound, at the indicated time points (see statistical results in Table SM1). * denotes p<0.05.

**Supplementary Figure 2.** Effects of pentedrone, pentylone, 4-MPD, NEPD, NEP and 4-MeAP on transport-mediated release of preloaded radiolabelled substrate from HEK293 cells stably expressing DAT. As indicated, after 6 min of basal release (either in the presence of KHB or KHB with monensin 10 µM), the compound of interest was added at 10 µM. Synthetic cathinones were grouped in pairs accordingly to the presence of a methyl or ethyl group at the amino terminal (pentedrone and NEPD; pentylone and NEP; 4-MPD and 4-MeAP). The effects of each synthetic cathinone in the presence of KHB or KHB with monensin 10μM are shown as empty or filled symbols, respectively. Data are mean ± SEM for n=3 experiments performed in duplicate. Data was statistically analyzed using with a mixed-effects model, employing Šidák’s correction for multiple comparisons. This statistical analysis explored possible significant differences between KHB + compound and KHB with monensin 10µM + compound, at the indicated time points (see statistical results in Table SM1).
